# Molecular detection of zoonotical *Giardia duodenalis*, *Cryptosporidium* spp. and *Blastocystis* in wild mesocarnivores from Eastern Spain

**DOI:** 10.3389/fvets.2025.1736482

**Published:** 2026-01-27

**Authors:** Alba Martí-Marco, Samantha Moratal, Irene Torres-Blas, Jesús Cardells, Víctor Lizana, María Auxiliadora Dea-Ayuela

**Affiliations:** 1 Servicio de Análisis, Investigación y Gestión de Animales Silvestres (SAIGAS), Facultad de Veterinaria, Universidad Cardenal Herrera-CEU, CEU Universities, Alfara del Patriarca, Spain; 2 Wildlife Ecology & Health Group (WE&H), Faculty of Veterinary, Universitat Autònoma de Barcelona (UAB), Bellaterra, Spain; 3 Departmento de Farmacia, Facultad de Ciencias de la Salud, Universidad Cardenal Herrera-CEU, CEU Universities, Alfara del Patriarca, Spain

**Keywords:** Blastocystis, *Cryptosporidium* spp., enteric parasites, *Giardia duodenalis*, human–wildlife interface, wild mesocarnivores, zoonotic transmission

## Abstract

**Introduction:**

Changes in land use and urbanization have altered the distribution and behaviour patterns of wildlife, increasing contacts between people and wild carnivores, elevating the risk of disease transmission. Evidence of enteric parasite presence in wild mesocarnivores from Spain is scarce, particularly in the eastern region.

**Methods:**

We surveyed 221 fecal samples collected in 2018–2023 from nine mesocarnivore species across 85 municipalities in the Valencian Community (eastern Spain). Molecular assays were used to detect *Giardia duodenalis*, *Cryptosporidium* spp, and *Blastocystis*, and positives were characterized by genetic sequencing when possible.

**Results:**

Overall prevalences were 6.8 % for *G. duodenalis* and for *Cryptosporidium* (15 of 221), and 8.6 % for *Blastocystis* (19 of 221). *G. duodenalis* was detected in seven of nine species, *Cryptosporidium* in four, and *Blastocystis* in six. Co-infections occurred but any sample harbored all three parasites. Sequencing revealed multiple *Cryptosporidium* species with relevance for humans and wildlife (including *C. meleagridis*, *C. canis*, *C. ditrichi*, *C. erinacei*, *C. muris*, and *C*. sp mouse genotype II), and *Blastocystis* subtypes 3, 4, 5, 6, 7, and 15 in several hosts. As in other studies, genotyping of *G. duodenalis* was unsuccessful.

**Discussion:**

Detection of prey-associated *Cryptosporidium* in predators supports trophic transmission. The presence of those zoonotic enteroparasites in wild mesocarnivores highlight the need for integrated wildlife and public health surveillance at the human–wildlife interface and for further work to resolve parasite sources, transmission pathways, and the conditions that facilitate cross-species spread.

## 1 Introduction

Over the last few decades, the coexistence dynamics between wild carnivores and people have changed significantly. Multiple studies indicate that wild mesocarnivores (e.g., red fox, stone marten, genet) are increasingly present near human settlements in Mediterranean landscapes, including Eastern Spain. This trend is driven by habitat fragmentation, urbanization, and land-use changes, which push these adaptable species into closer proximity with people and domestic animals (1–5). Camera trap studies and ecological surveys show that generalist mesocarnivores are more abundant in urban and peri-urban areas (2, 3, 5). This phenomenon has led to a significant increase in interactions between humans and wildlife (2, 6, 7) with a consequent increase in the risk of disease transmission between them (4, 8, 9).

*Giardia duodenalis*, *Cryptosporidium* spp. and *Blastocystis* are some of the most prevalent zoonotic enteroparasites found in both human and animal feces (9–12). These unicellular parasites typically cause gastrointestinal disorders, such as diarrhea, which is more severe in either young or immunocompromised individuals (13–16). Environmental prevalence of these parasites has increased in certain regions of Spain, including Eastern areas (17) where climatic conditions such as milder winters and higher precipitation levels may favour the survival of environmental stages (e.g., cysts and oocysts) that are resistant to desiccation and temperature extremes (18). Recent studies have also indicated higher parasite loads in wildlife populations inhabiting these areas, suggesting an increasing environmental prevalence that might exacerbate zoonotic risks (19).

In Spain, several epidemiological and molecular studies have analyzed the presence, as well as the species, genotypes, or subtypes of these enteroparasites in different wildlife taxa, such as ungulates (19–22), birds (23) or rodents (24). However, there is little information available regarding the role that wild mesocarnivores play as these parasites’ hosts both at national and European levels, despite mesocarnivores often interacting with a variety of other wildlife and human-modified environments, creating multiple potential transmission routes for zoonotic pathogens by attacking humans or domestic animals; or defecating in public spaces (25, 26). The ecology and behaviour of mesocarnivores, who often act as scavengers, have big home ranges, high mobility and are quite adaptable to urban and peri-urban areas increase their likelihood of encountering contaminated water, prey, or environments, which can elevate exposure to *Giardia* and *Cryptosporidium* (27). For example, red foxes, due to their adaptability and presence in human-modified landscapes, are repeatedly identified as suitable hosts for zoonotic *Giardia* and *Cryptosporidium* species (27). Furthermore, red foxes are classified as a game species in Spain, meaning that both hunters and hunting dogs can have direct contact with animals that can be asymptomatic carriers through contact with the carcass or direct ingestion of the contaminated gastrointestinal System in case of the hunting dogs. (25, 26, 28–30). This, coupled with high human population density and changes in human spatial use, could heighten wildlife-to-human parasite transmission (19, 31). The very low infective dose (13) and high resistance of these parasites in the environment and to conventional water treatments (16) increase transmission risk.

Some studies have been conducted in different areas of Spain (25, 26, 28, 29), but none of them cover the eastern region of the country, where climatic conditions could favor the survival of resistance forms of these parasites (30).

Given the significance of these enteroparasites for both human and animal health, and in light of the increasing interactions between people and wildlife, the main objective of this study is to assess the presence of these parasites in wild mesocarnivores found in Eastern Spain, as well as their circulation among the nine species selected as potential carriers.

## 2 Materials and methods

### 2.1 Study area and sample selection

**Figure 1:**
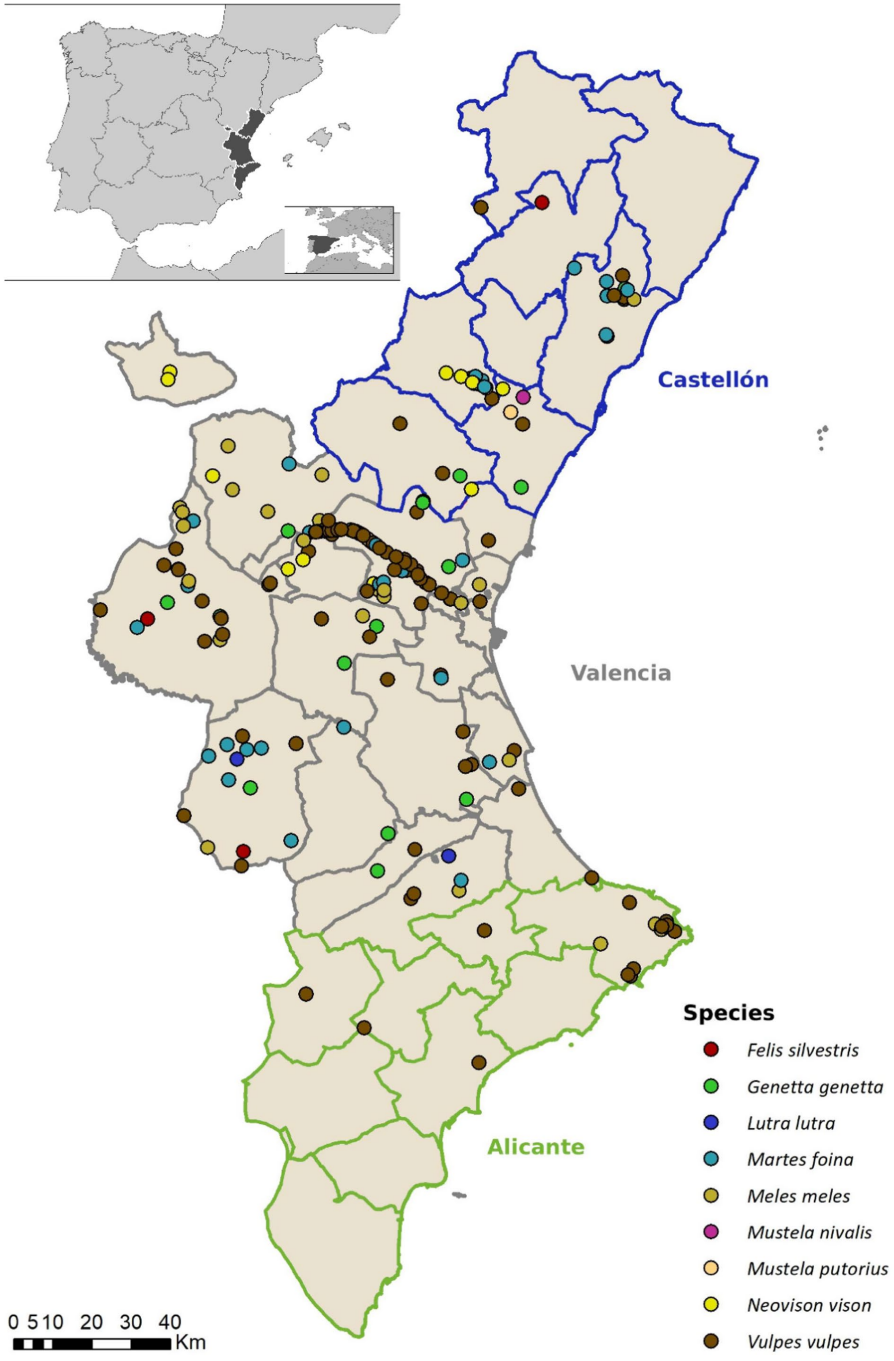
Spatial distribution of collected samples in the Valencian community (Eastern Spain), categorized by mesocarnivore species.

The sampling area is comprised of a total of 85 municipalities belonging to Valencian Community (Spain). All the municipalities included in the study have confirmed the presence of wild mesocarnivore species within their territory (32) (Figure 1).

Sample size (N) was calculated with WinEpi 2.0 software (33). The red fox was the chosen species to calculate sample size. There is literature available regarding the prevalence of both *Giardia duodenalis* and *Cryptosporidium* spp. in this species in other areas of Spain and other European countries (26, 34–37). Prevalence values in this literature ranged from 4.8% to 9.6 for *Giardia duodenalis* and from 2.2 to 8% for *Cryptosporidium* spp. Therefore, we used expected prevalence values of 9.6 and 8%, respectively, to calculate sample size. Assuming an unknown population, the 95% of confidence level and 5% of error (26, 34–37). Sample collection was performed from January 2018 to January 2023. We collected a total of 221 samples from nine different species: red fox (*Vulpes vulpes*, *N =* 121), stone marten (*Martes foina*, *N =* 31), Eurasian badger (*Meles meles*, *N =* 30), common genet (*Genetta genetta*, *N =* 18), American mink (*Neovison vison*, *N =* 13), Eurasian otter (*Lutra lutra*, *N =* 2), European wildcat (*Felis silvestris*, *N =* 4), European polecat (*Mustela putorius*, *N =* 1) and least weasel (*Mustela nivalis N =* 1). The animals included in the study were roadkills, hunted (some of the red foxes), derived from eradication programs (American mink, considered as an invasive species in Europe) or from local wildlife rescue centers. We also recorded location, sex, age and weight of the sampled individuals. Sex was determined by assessment of external genitalia and age was divided into three categories: cub, juvenile and adult.

Fecal samples were collected directly from the rectum and stored in 50 mL sterile polypropylene screw-top containers. Each container was properly labeled with the identification number for each animal. Samples were kept at 4 °C and processed within the next 24 h post-collection at School of Veterinary Medicine (Universidad CEU Cardenal Herrera, Valencia, Spain). Sample DNA was extracted and stored at −20 °C until the molecular analysis was performed.

### 2.2 DNA extraction

DNA extraction was performed using NZY Tissue gDNA Isolation kit (Nzytech genes & enzymes, Lisboa, Portugal) according to the manufacturer’s instructions.

#### 2.2.1 *Giardia duodenalis* detection

A qPCR for *G. duodenalis* detection was performed using an adapted form of the protocol described by Dacal et al. (38) and Verweij et al. (39), targeting a 62 bp segment of the SSU rRNA gene. A total of 3 μL of DNA per sample were used in a total volume of 25 μL for PCR running. The PCR pre-mixture included 12.5 pmol of both Gd-80F and Gd-127R primers (39), 10 pmol for the probe (Table 1), and 12.5 μL of NZY Supreme qPCR Probe Master Mix (Nzytech genes & enzymes, Lisboa, Portugal). The AriaMx (Agilent Technologies, Santa Clara, CA, USA) system was used for parasite DNA detection.

**Table 1:** Oligonucleotides employed for the molecular identification and/or characterization of *Giardia duodenalis*, *Cryptosporidium* spp., and *Blastocystis.*

Organism	Gen	Oligonucleotides	Sequence (5′–3′)	References
*Giardia duodenalis*	SSU rRNA	Probe	FAM– CCCGCGGCGGTCCCT GCTAG–BHQ1	(39)
		Gd-80F	GACGGCTCAGGACAA CGGTT	(39)
		Gd-127R	TTGCCAGCGGTGTCCG	(39)
	*gdh*	GDHeF	TCAACGTYAAYCGYG GYTTCCGT	(38)
		GDHiF	CAGTACACCTCYGCTC TCGG	(38)
		GDHiR	GTTRTCCTTGCACATC TCC	(38)
	*βg*	G7 F	AAGCCCGACGACCTC ACCCGCAGTGC	(41)
		G759 R	GAGGCCGCCCTGGAT CTTCGAGACGAC	(41)
		G376_F	CA-TAACGACGCCATCGC GGCTCTCAGGAA	(79)
*Cryptosporidium* spp.	SSU rRNA	18SicF2	GACATATCATTCAAGTTTCTGACC	(42)
		18SicR2	CTGAAGGAGTAAGGAACAACC	(42)
		18SicF1	CCTATCAGCTTTAGACGGTAG	(42)
		18SicR1	TCTAAGAATTTCACCTCTGACTG	(42)
*Blastocystis*	SSU rRNA	BL18SR2PP BL18SPPF1	AGTAGTCATACGCTCGTCTCAAA	(44)

Amplification consisted of 15 min at 95 °C, followed by 45 amplification cycles of 15 s at 95 °C and 1 min at 60 °C (38). Both positive (*G. duodenalis* genotype type C isolated from an infected dog) and negative controls were included in each PCR run.

Samples positive to qPCR were subsequently assessed by using two semi-nested PCRs which allowed further genotyping of the genes encoding for the glutamate dehydrogenase (*gdh*) and *β*-giardin (*bg*) proteins of the enteroparasite. Briefly, a 432 bp fragment from the gdh gene was amplified using a PCR reaction mixture which contained 3 μL of sample DNA in a total final volume of 25 μL, which included 1 μL of primer pairs GDHeF/GDHiR in the primary reaction; and 1 μL of the primary reaction in a total volume of 25 μL, which included 1 μL of primer pairs GDHiF/GDHiR in the secondary reaction (Table 1) (38). Both amplification PCR procedures were run in a GeneAmp PCR System 2,700 thermocycler (Applied Biosystems, Foster City, CA, USA). The protocol used to carry out the *ghd* gene amplification started with a denaturation process at 95 °C for 3 min, followed by 35 amplification cycles of 95 °C for 30 s, 55 °C for 30 s, and 72 °C for 1 min, with a final extension of 72 °C for 7 min (38, 40). All PCR reactions included positive and negative controls.

Similarly, for the *bg* gene, a 753 bp gene fragment was amplified using 3 μL of the sample DNA in a total volume of 25 μL, which included 1 μL of the primer pairs G7/G759R for the primary reaction (Table 1). For the secondary PCR reaction, 1 μL of the primary reaction was used in a total volume of 25 μL, which included 1 μL of the primer pairs G376/G759 (41), following the protocol described by Mahbubani et al. (1992a). Both PCR amplification reactions were performed using a GeneAmp PCR System 2,700 thermocycler (Applied Biosystems, Foster City, CA, USA). The protocol described by Dacal et al. (38), consisted of a first denaturation step of 95 °C for 7 min, followed by 35 amplification cycles of 95 °C for 30 s, 65 °C for 30 s, and 72 °C for 1 min, with a final extension of 72 °C for 7 min Positive and negative PCR controls were included in all the reactions.

Semi-nested PCR results were visualized in a 1.5% agarose gel pre-stained with RedSafe TM nucleic acid dye (iNtRON Biotechnology, Seongnam, Republic of Korea).

#### 2.2.2 *Cryptosporidium* spp. detection

The presence of *Cryptosporidium* spp. was evaluated using a nested PCR, and a 587 bp SSU rRNA gene fragment was amplified, following the protocol described by Ryan et al. (42). The PCR reaction was carried out using 3 μL of DNA sample, in a total final volume of 25 μL, which included 12.5 pmol of each of the forward primer 18SicF2 and reverse primer 18SicR2 for the first reaction, and forward primer 18SicF1 and reverse primer 18SicR1 for the second reaction (Table 1).

Both amplification reactions were carried out in a GeneAmp PCR System 2,700 (Applied Biosystems, Foster City, CA, USA) thermocycler, using the conditions described by Ryan et al. (42): a first denaturation step of 95 °C for 5 min, followed by 45 amplification cycles (30 s at 94 °C, 30 s at 58 °C, 30 s at 72 °C) and a final extension process that lasted 10 min at 72 °C. For all the PCR reactions, negative and positive (sample from a farm positive to *Cryptosporidium ubiquitum*) controls were included. Positive samples were visualized in a 1.5% agarose gel pre-stained with RedSafe Tm (iNtRON Biotechnology, Seongnam, Republic of Korea) nucleic acid stain.

#### 2.2.3 *Blastocystis* detection

The presence of *Blastocystis* was evaluated using a conventional PCR protocol adapted from Gantois et al. (43), employing a SSU rRNA gene fragment of 320–342 bp. For each PCR reaction, 2 μL of sample DNA were added in a total volume of 50 μL, which included 0.5 μL of primers BL18SPPF1 and BL18SR2PP (Table 1) (44) and the Master Mix Supreme NZYTaq II 2x Green Master Mix (Nzytech genes & enzymes, Lisboa, Portugal).

The PCR amplification reaction was conducted in a GeneAmp PCR System 2,700 thermocycler (Applied Biosystems, Foster City, CA, USA) using the settings described by Gantois et al. (43): a first denaturation step at 95 °C for 5 min, followed by 40 amplification cycles (30 s at 94 °C, 35 s at 60 °C, 50 s at 68 °C), and a final extension step at 68 °C for 2 min. Positive and negative controls were included in all PCR reactions (samples from domestic pigs positive for *Blastocystis* ST5). Positive samples were visualized in a 1.8% agarose gel pre-stained with Greensafe Premium nucleic acid stain (Nzytech genes & enzymes, Lisboa, Portugal).

### 2.3 Phylogenetic analysis and sequentiation

Positive samples that showed a band of the expected size were sequenced by an external sequencing service (Genomics Department from Centro de Investigación Príncipe Felipe, Valencia, Spain). The nucleotide sequences obtained were visualized using Chromas software version 2.6.6 (Technelsyum DNA Sequencing Software, South Brisbane, QLD, Australia) and compared using the online BLAST tool (Basic Local Alignment Search Tool)^1^ with the sequences available for *G. duodenalis*, *Cryptosporidium* spp. and *Blastocystis* sp. in the NCBI GenBank database. Alignments with reference sequences were carried out using the MEGA program, version X (45).

### 2.4 Statistical analysis

A series of generalized linear models (GLMs) using the presence/absence of each studied parasite (*Giardia duodenalis*, *Cryptosporidium* spp., and *Blastocystis* sp.) as the response variable were performed. The explanatory variables included in each model were all the same for each parasite, and were host age class (Juvenile, Sub-Adult, Adult), Province (Valencia, V; Alicante, A; Castellón, C; unknown, X), and sample origin (Capture and release; disease; drowned; found dead; hunted; roadkill; alive animal; anthropogenic cause and fresh feces). Factor levels with low sample sizes (N < 10) were pooled to avoid model separation and overparameterization. Model assumptions and goodness of fit were assessed using simulation-based residual diagnostics implemented in the DHARMa Package (46). Uniformity of residuals, dispersion, and potential deviations from model expectations were evaluated using Kolmogorov–Smirnov and quantile tests. No evidence of overdispersion or global lack of fit was detected. Minor deviations in lower conditional quantiles were observed, which are expected for binomial models applied to low-prevalence data. Post-hoc comparisons among factor levels were conducted if necessary, using estimated marginal means (EMMs) with pairwise contrasts implemented in the *emmeans* Package (47), based on the fitted binomial GLMs. Comparisons were adjusted for multiple testing using Tukey’s method.

## 3 Results

This study included a total of 221 samples, belonging to nine different wild mesocarnivore species found in Valencian Community. We detected *G. duodenalis* in seven species: *V. vulpes, M. foina, N. vison, M. meles, G. genetta, L. lutra* and *F. silvestris*; *Cryptosporidium* spp. in four: *V. vulpes, N. vison, M. meles, G. genetta*; and *Blastocystis* sp. in six: *V. vulpes, M. foina, M. meles, G. genetta, M. putorious* and *M. nivalis*. Prevalences of *G. duodenalis, Cryptosporidium* spp. and *Blastocystis* were 6.79, 6.79, and 8.59%, respectively. Table 2 shows parasite distribution according to species and sex.

**Table 2:** Detected prevalence of *Giardia duodenalis*, *Cryptosporidium* spp., and *Blastocystis* in wild mesocarnivores according to species and sex.

Host species	Sex	*Giardia duodenalis*	*Cryptosporidium* spp.	*Blastocystis*
*Vulpes vulpes*	M	1.52% (1/66)	9.09% (6/66)	10.61% (7/66)
	F	6.52% (3/46)	8.70% (4/46)	8.70% (4/46)
	ND	11.11% (1/9)	0% (0/9)	11.11% (1/9)
	**Total**	**4.13% (5/121)**	**8.26% (10/121)**	**9.91% (12/121)**
*Martes foina*	M	0% (0/18)	0% (0/18)	0% (0/18)
	F	25% (2/8)	0% (0/8)	12.5% (1/8)
	ND	20% (1/5)	0% (0/5)	0% (0/5)
	**Total**	**9.68% (3/31)**	**0% (0/31)**	**6.45% (2/31)**
*Meles meles*	M	0% (0/11)	0% (0/11)	0% (0/11)
	F	7.14% (1/14)	14. 29% (2/14)	7.14% (1/14)
	ND	0% (0/5)	0% (0/5)	0% (0/5)
	**Total**	**3.33% (1/30)**	**6.66% (2/30)**	**3.33% (1/30)**
*Genetta genetta*	M	18.18% (2/11)	9.09% (1/11)	9.09% (1/11)
	F	14.29% (1/7)	0% (0/7)	14.29% (1/7)
	**Total**	**16.67% (3/18)**	**5.56% (1/18)**	**11.11% (2/18)**
*Neovison vison*	M	16.67% (1/6)	33.33% (2/6)	0% (0/6)
	F	0% (0/7)	0% (0/7)	0% (0/7)
	**Total**	**7.69% (1/13)**	**15.38% (2/13)**	**0% (0/13)**
*Lutra lutra*	M	0% (0/1)	0% (0/1)	0% (0/1)
	ND	100% (1/1)	0% (0/1)	0% (0/1)
	**Total**	**50% (1/2)**	**0% (0/2)**	**0% (0/2)**
*Felis silvestris*	M	0% (0/2)	0% (0/2)	0% (0/2)
	F	100% (1/1)	0% (0/1)	0% (0/1)
	ND	0% (0/1)	0% (0/1)	0% (0/1)
	**Total**	**25% (1/4)**	**0% (0/4)**	**0% (0/4)**
*Mustela putorius*	H	0% (0/1)	0% (0/1)	100% (1/1)
	**Total**	**0% (0/1)**	**0% (0/1)**	**100% (1/1)**
	M	0% (0/1)	0% (0/1)	100% (1/1)
*Mustela nivalis*	**Total**	**0% (0/1)**	**0% (0/1)**	**100% (1/1)**

M: male; F: female; ND: No data available. Bold values are highlight the data corresponding to the total data for each species of mesocarnivore.

We did not find any sample positive to all three enteroparasites. However, we found co-infections between *G. duodenalis* and *Cryptosporidium* spp. in red fox (*N =* 1) and a common genet (*N =* 1); *G. duodenalis* and *Blastocystis* in a common genet (*N =* 1) and finally, *Cryptosporidum* spp. and *Blastocystis* in a red fox (*N =* 1).

**Figure 2:**
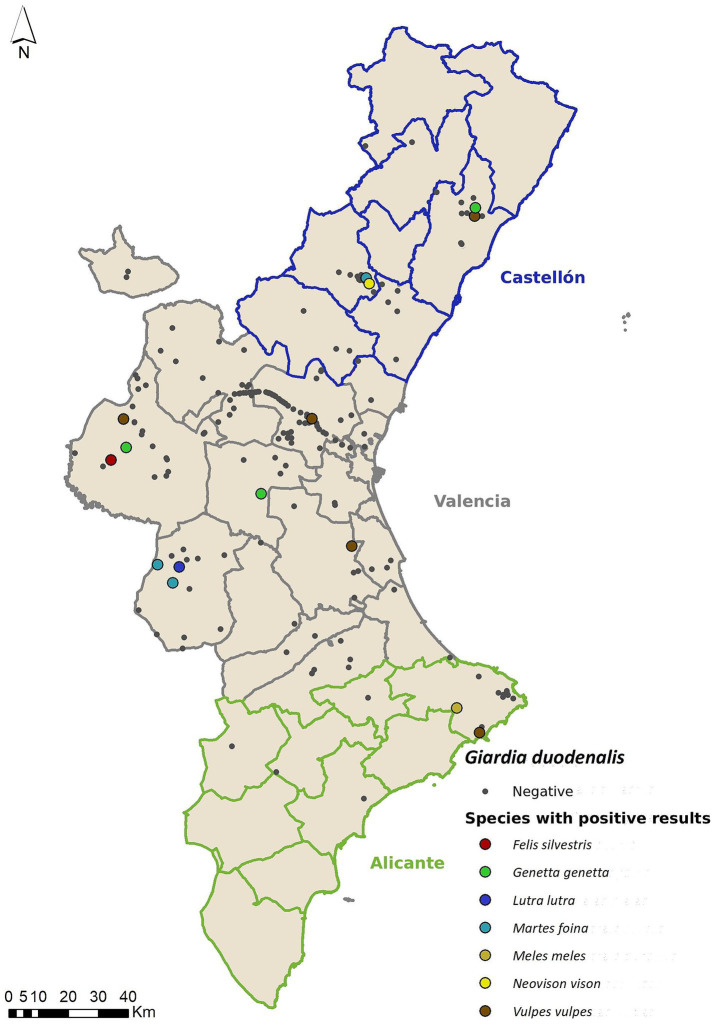
Geolocation of wild mesocarnivores testing positive for *Giardia duodenalis*, categorized by host species.

*G. duodenalis* was the most widely distributed parasite among the studied mesocarnivore species, being found in seven out of a total of nine (Figure 2). General *G. duodenalis* prevalence was 6.79% (15/221; CI 95% 3.5% – 10.1%). Mean Ct value was 36.03 (range: 29–39.6). As in previous studies (21, 22, 24, 26, 48) genotyping of positive samples/isolates was unsuccessful; therefore, we could not determine the genovariety to which they belonged. None of the explanatory variables included in the GLM model were significant (Table 3).

**Table 3:** GLM results for each studied parasite model.

Parasite	*Giardia duodenalis*						*Cryptosporidium* spp				*Blastocystis* sp			
Explanatory variables	Categories	*N*	Intercept	SE	z-value	*p*-value	Intercept	SE	z-value	*p*-value	Intercept	SE	z-value	*p*-value
Age	J	8	−16.603	2058.36	−0.008	> 0.05	−17.037	3462.141	−0.005	> 0.05	0.038	1.201	0.032	> 0.05
	SA	29	−1.144	1.08	−1.055	> 0.05	1.556	0.716	2.173	**0.03**	0.144	0.729	0.197	> 0.05
Province	C	26	−0.172	1.21	−0.142	> 0.05	19.136	2368.902	0.008	> 0.05	−1.610	0.969	−1.661	> 0.05
	V	138	−0.818	1.14	−0.720	> 0.05	19.274	2368.902	0.008	> 0.05	−3.121	0.909	−3.345	**< 0.001**
	X	4	−16.342	3041.31	−0.005	> 0.05	21.676	2368.903	0.009	> 0.05	−1.382	1.483	−0.932	> 0.05
Origin	Hunted	15	−0.188	1.53	−0.123	> 0.05	2.755	1.517	1.816	> 0.05	−3.221	1.568	−2.055	**0.04**
	Other	13	1.505	1.23	1.221	> 0.05	−16.755	2841.426	−0.006	> 0.05	−0.020	1.167	−0.018	> 0.05
	Roadkill	144	−0.783	1.13	−0.690	> 0.05	0.016	1.198	0.013	> 0.05	−0.908	0.880	−1.014	> 0.05

The intercept included the values of age: adult (*N =* 147), province: Alicante (A, *N =* 16) and origin: anthropogenic (*N =* 12). N: Sample size; J: juvenile; SA: sub-adult. C: Castellón; V: Valencia; X: unknown province. SE: Standard error. In the variable “Origin,” the category “Other” is the result of pooling all levels with N < 10, which include “Capture-release,” “Alive,” “Pit,” “Drowned” and “Disease.” Significant *p*-values are marked in bold.

**Figure 3:**
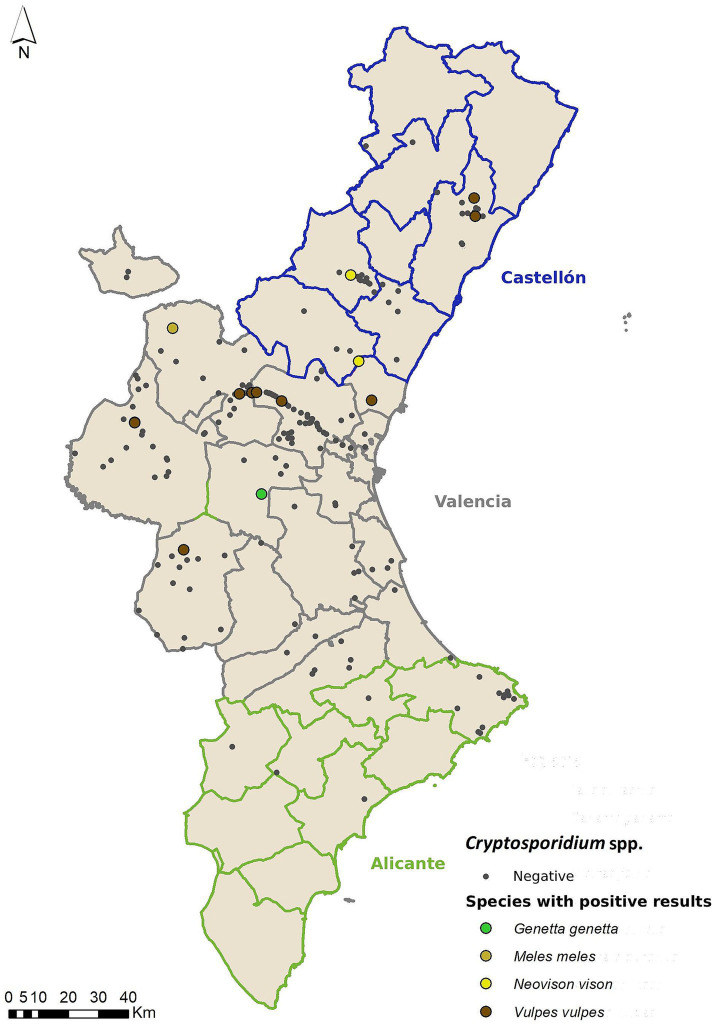
Geolocation of wild mesocarnivores testing positive for *Cryptosporidium* spp., categorized by host species.

Prevalence of *Cryptosporidium* spp. was 6.79% (15/221; CI 95% 3.5–10.1%) and was found in four mesocarnivore species (Figure 3). Genetic sequencing revealed that different species of *Cryptosporidium* spp. were present in the samples, with the full list shown in Tables 4, 5. The partial sequences of *Cryptosporidium* spp. obtained, were deposited in GenBank under the following accession numbers: PP002598 - PP002601. Only the variable “Age” was statisically significant (*p =* 0.03), with the differences being found between adults (prevalence 0.05%) and sub-adults (Prevalence 0.13%, *p =* 0.007). There were no other significant differences among the other categories.

**Table 4:** Sequence analysis results of *Cryptosporidium* spp. in wild mesocarnivores from the Valencian community.

Host species	*N*	Positive samples	Molecular characterization
*Vulpes vulpes*	121	10	*C. canis* (8/10) *C.* sp. mouse genotype II (1/10) *C. meleagridis* (1/10)
*Neovison vison*	13	2	*C. mink* (2/2)
*Meles meles*	30	2	*C. ditrichi* (1/2) *C. erinacei* (1/2)
*Genetta genetta*	18	1	*C. muris* (1/1)

N: Sample size.

**Table 5:** Isolates of *Cryptosporidium* spp. classified by species according to the nearest reference sequence.

Host species	*N*	Reference sequence	% ID	SNV	Accession number
*C. canis*	2	KU608308.1	100	None	
	3	KU608308.1	99,81	G➔A	PP002598 PP002600 PP002601
	3	MT329018.1	100	None	
*C. ditrichi*	1	MN065795.1	98,64	2 GAP	PP002599
*C. erinacei*	1	OQ109275.1	100	None	
*C. meleagridis*	1	MN410718.1	100	None	
*C. mink genotype*	2	MN235855.1	100	None	
*C. muris*	1	MK457349.1	100	None	
*C.* sp. mouse genotype II	1	EF546483.1	100	None	

N: Sample size; % ID: percentage of identity; SNV: single nucleotide variant.

**Figure 4:**
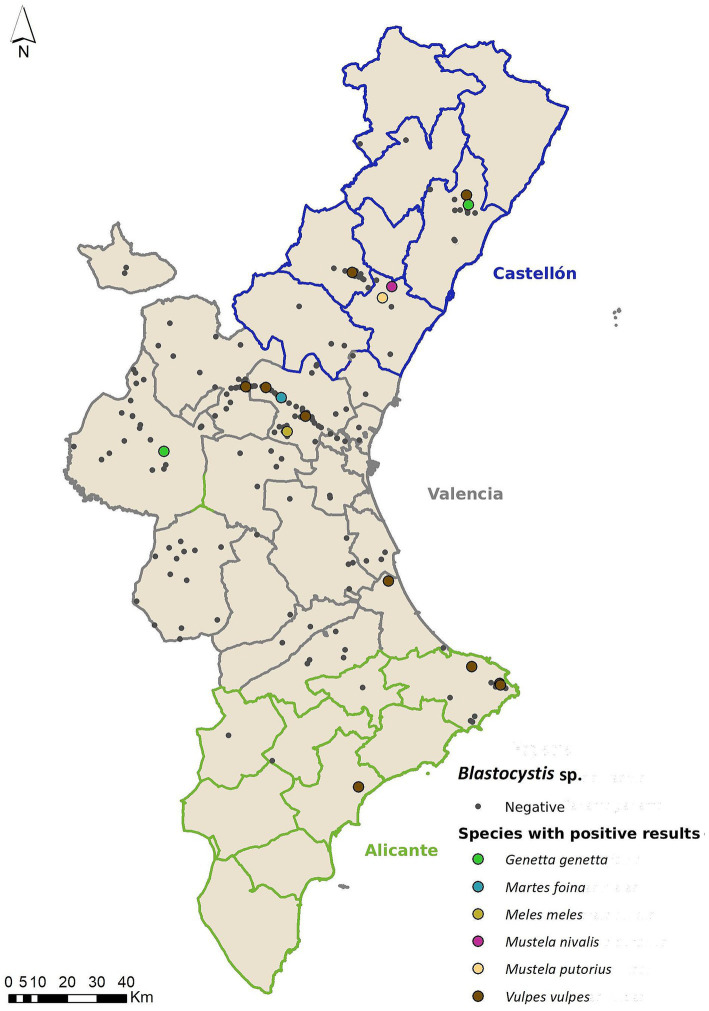
Geolocation of wild mesocarnivores testing positive for *Blastocystis*, categorized by host species.

*Blastocystis* was the enteric pathogen with the highest prevalence found in this study, with 8.59% (19/221; CI 95% 5.26–13.10% %), being detected in six out of the nine analyzed species (Figure 4). The genetic sequencing revealed a wide range of *Blastocystis* subtypes (ST) as shown in Tables 6, 7. The partial sequences of *Blastocystis* obtained were deposited in GenBank under the numbers: PP002606 - PP002613. Regarding *Blastocystis,* only the variables “Province” (*p* < 0.001) and “Origin” (*p =* 0.04) were significant. Valencia was significantly different from Alicante (*p =* 0.003), and marginally different from Castellón (*p =* 0.08). Prevalence values for *Blastocystis* on these provinces were 7.55% (4/53) in Alicante; 19.23% (5/26) in Castellón; 5.07% (7/138) in Valencia.

**Table 6:** Sequence analysis results of *Blastocystis* in wild mesocarnivores from the Valencian community.

Host species	*N*	Positive samples	Molecular characterization
*Genetta genetta*	18	2	ST4 (1/2) ST15 (1/2)
*Martes foina*	31	2	ST5 (1/2) ST15 (1/2)
*Meles meles*	30	1	ST5 (1/1)
*Mustela nivalis*	1	1	ST5 (1/1)
*Mustela putorius*	1	1	ST5 (1/1)
*Vulpes vulpes*	121	12	ST15 (2/12) ST3 (1/12) ST5 (5/12) ST6 (1/12) ST7 (3/12)

N: Sample size.

**Table 7:** Isolates of *Blastocystis* classified by species according to the nearest reference sequence.

Host species	*N*	Reference sequence	% ID	SNV	Accession number
*Blastocystis* ST15	5	MK801422.1	100	None	
*Blastocystis* ST3	1	KP055739.1	100	None	
*Blastocystis* ST4	1	MH127500.1	99.65	A➔G	PP002610
*Blastocystis* ST5	4	MK801419.1	100	None	
	4	MK801419.1	99.64–99.66	G➔A	PP002606 PP002607 PP002608 PP002609
*Blastocystis* ST6	1	MT039591.1	100	None	
*Blastocystis* ST7	1 1 1	MT039562.1 MT039562.1 OQ571598.1	99.65 99.64 99.31	A➔T T➔C T➔A (x2)	PP002611 PP002613 PP002612

N: Sample size % ID: percentage of identity; SNV: single nucleotide variant.

## 4 Discussion

The number of studies describing the presence and prevalence of *Giardia duodenalis*, *Cryptosporidium* spp. and *Blastocystis* in wild carnivore species found in Spain is very limited. To the authors’ knowledge, this is the first study conducted in Eastern Spain evaluating the presence of these three enteric parasites in wild mesocarnivore species, and we have found a prevalence of 6.79% for both *G. duodenalis* and *Cryptosporidium* spp. and 8.59% for *Blastocystis*.

The observed prevalence for *G. duodenalis* (6.79%) is similar to other studies conducted in the Iberian Peninsula in different carnivore species: 4.7% in a country study involving several regions (Andalusia, Asturias, Basque Country, Castilla – La Mancha and Extremadura) (26) and 9.6% in Northwestern Galicia (34). However, other available research carried out in the Iberian Peninsula yields heterogenic results, by presenting higher values in areas from Portugal (49) [15.8% of prevalence in red foxes and 18.6% in stone marten (*M. foina*) (49)], and significantly lower values within other territories of Spain [only 1 positive stone marten out of 193 samples from 11 mesocarnivore species (26)]. A prevalence of 6.8% (30/347) in Eurasian otters (*L. lutra*) has been reported from Galicia (NW Spain) (29). In our study we only had two otter samples, with one of them being positive. The wide distribution of both *Giardia* spp. and *Cryptosporidium* spp. in water bodies and even tap water across Galicia and other Northern areas of Spain has been proven in the past (50, 51), showcasing the wide environmental distribution of these pathogens whose main infective pathways are food-borne and water-borne. These results regarding the positive samples fund in otters suggest that river water could be highlighted as a possible source of infection for this mammal species, since aquatic ecosystems possess environmental conditions that favor the survival of *Giardia* resistance forms (52), potentially indicating that infection in otters may follow the same mechanism as for humans.

As previously described in the results section, we detected *G. duodenalis* in seven (out of nine) mesocarnivore species, with marked differences in prevalence levels between them (although we have to consider the discrepancies among sample sizes for each species): red fox (4.13% - 5/121), stone marten (9.68–3/31%), American mink (7.69% - 1/13), Eurasian badger (3.33% - 1/30), common genet (16.67% - 3/18), Eurasian otter (50% - 1/2) and European wildcat (25% - 1/4). Among all of them, the red fox is the most studied species in the European context for the three target parasites of the present work. The red fox is the only one of these mesocarnivores that is categorized as a hunting species, making sampling acquisition easier. Prevalence rates for *G. duodenalis* in the red fox are reported to be 4.8% in Norway (35), 4.6% in Romania (53), 4.5% in Croatia (54) and 19% in Poland (55). One study in Sweden found a noteworthily high prevalence of 44%, after detection using direct immunofluorescence techniques (56). These marked discrepancies among prevalence results could stem from the different methodologies used for detection; nevertheless, it might suggest that there are significant differences regarding parasite distribution between bioregions/areas/countries.

The red fox has been one of the most researched species thanks to its ability to adapt to highly anthropized environments due to its dietary and habitat plasticity. The Valencian Community hosts a great diversity of species with overlapping habitat ranges, therefore facilitating the transmission of *G. duodenalis* among them. Our prevalence values in red fox are low, suggesting that zoonotic transmission risk of these parasites from this mesocarnivore species is minimal. However, molecular studies are practically nonexistent at both national and European levels (10).

In Western Europe, the most relevant study to date that has detected *G. duodenalis* was carried out by Maestrini et al. (57), obtaining a prevalence of 48.8% by immunoassay in Eurasian badgers from an anthropized area in central Italy. In our study, we only found one positive Eurasian badger (out of 30) (Figure 2). Most of our Eurasian badgers came from rainfed croplands, which are areas with low human population. Additionally, *G. duodenalis* is a parasite with a direct life cycle, normally transmitted feco-orally; its most typical infection route in human beings is through the ingestion of untreated infected water carrying cysts. The high prevalence results obtained in Italy compared to our low prevalence could therefore stem from differences in the regional climate; the low human population density found in rainfed croplands and finally from differences in laboratory methodologies, as the results from Italy are reported from immunoassay results (which measure exposure to the parasite); compared to our molecular approach to determine presence of *G. duodenalis.*

*G. duodenalis* is rarely reported from other continents. In Philippines, a study involving captive specimens of palm civets (*Paradoxurus hermaphroditus*) and binturong (*Arctictis binturong whitei*) also documented the presence of this parasite (58). It is note-worthy, though, that the sampled animals came from captive conditions, which inherently implies a close contact with their keepers, again potentially suggesting that transmission may occur from humans to wildlife. This highlights the results of the present work, with the detection of 6.8% prevalence and involving seven of the nine species sampled, showing that, despite having low parasite prevalence values, *Giardia duodenalis* is quite widespread among mesocarnivore populations from Eastern Spain.

None of our samples could be genotyped successfully using a semi-nested PCR for *gdh* and *βg*. Unfortunately, genotyping failure is a recurring problem in studies focused on *G. duodenalis* detection (21, 22, 24, 26, 48). Future studies could enhance the molecular characterization of *Giardia duodenalis* by incorporating additional genetic loci to support a comprehensive multilocus genotyping approach, such as tpi or ef1-*α*.

Regarding *Cryptosporidium* spp., the prevalence levels observed in this work (6.79%) are similar to prevalence levels obtained in previous studies carried out in Spain and also involving several different mesocarnivore species (5.7%) (26). In Europe, Perec-Mastysiak et al. (59) obtained much higher prevalence levels in Poland through immunofluorescence techniques (23%). Given that the studies with higher prevalence come from works carried out using immunofluorescence techniques, this situation highlights the importance of the potential differences between the results of different detection methods.

There were significant differences between adult and sub-adult animals in prevalence levels in our study, with sub-adult animals presenting a lower prevalence (1.8%, 4/221) compared to adults (3.62%, 8/221). These results are contradictory to the available literature, as younger animals generally show higher prevalence rates (18, 60, 61), especially for zoonotic *Cryptoposporidium* spp. such as *C. parvum* (62). However, the different age thresholds where changes in prevalence can happen can be species- specific. All the positive subadults and adults (except from one adult badger and one adult common genet) were foxes. One study (18) found that this prevalence pattern was different in the red fox, with the highest prevalence was found in animals older than 12 months compared to 5–6 months, but this difference was not statistically significant. These results are more in accordance with our observations. However, given the very low prevalence values obtained in this study, the statistical results need to be interpreted with caution.

Research conducted in only one mesocarnivore species showcased results such as those obtained by Méndez-Hermida et al. (29) in Northwestern Spain, finding a prevalence of 3.9% in Eurasian otter (*L. lutra*); or Gómez-Couso et al. (25), who found a prevalence of 24.2% in American mink (*N. vison*) in a study that included farmed minks and through immunofluorescence techniques. In the present study, *Cryptosporidium* spp. was not detected in any of the two otter specimens analyzed; in American mink, *Cryptosporidium mink* was detected in two of the 13 samples analyzed, so its presence is around 15.38%. This *Cryptosporidium* species has been detected in young people aged 11–21 years in Australia (63). These results differ from a recent study from England where it was detected in otter (2.17%) but not in American mink (64). Once again, terrestrial mammals which greatly rely upon aquatic environments seem to be more at risk of carrying these endoparasites; however, no solid conclusions can be reached on whether these species act as a source of endoparasite propagation or are infected through the same mechanisms as humans. In this sense, it would be pertinent to continue research on these species at the national and European levels to try to discern whether they are either reservoirs or sentinels.

We obtained a *Cryptosporidium* spp. prevalence of 8.26% in the red fox. The red fox is also the species where we detected the highest number of *Crypstosporidium* spp.: *C. canis*, *C.* sp. mouse genotype II and *C. meleagridis* (Table 4). Other studies also obtained similar prevalence levels in this species: 8% in a generalized study in several areas of Spain (26); 6.1% in Northwestern Spain (34); 3.4% in Portugal (49); 2.2% in Norway (35); 1.72 - 2.7% in Central Europe (65); and 12% in Poland (59). In contrast, in Ireland prevalence rised up to 20% (66) and, in Slovenia, up to 38.7% (67). This disparity in observed prevalences may be due to the methodology followed, both for sampling and detection techniques, with microscopy or immunochromatography being less sensitive than molecular techniques (65).

Regarding *Cryptosporidium* spp. diversity, the range of species previously detected in red foxes from Spain is quite wide: *C. hominis*, *C. canis*, *C. parvum*, *C. suis*, *C. felis* and *C. ubiquitum* (26, 34). At the European level, *C. tyzzeri, C. andersoni, C. galli* (65), *C. alticolis* and *C. vole* genotype II (59) have also been found. Some of these species, such as *C. hominis, C. parvum, C. ubiquitum* or *C. meleagridis* (detected in the present study) are zoonotic; others, like *C. suis* or *C. mink* have been sporadically found in humans (63, 68–70). Finally, the presence of *C.* sp. mouse genotype II can be explained by ingestion of infected mice, as they are part of red fox diet. This fact highlights the need for further study of hosts capable of propagating and facilitating the transmission of these parasite species.

As previously discussed, having access to samples of protected wild mesocarnivores species (all except the red fox, which is a game species, and the American mink, which is considered as an invasive species subject to eradication) can be a challenge. These sampling limitations likely contribute to having very little information available regarding the pathogens carried by these species. In the present study, *C. ditrichi* and *C. erinacei* were detected in two of the 30 Eurasian badger samples and *C. muris* in one of the 18 common genets, representing a presence of 6.66 and 5.56%, respectively (Table 4). In the case of Eurasian badgers in Spain, Mateo et al. (26) detected a prevalence of 3% and a single positive common genet (1/6, 16.67% prevalence). At the European level, Maestrini et al. (57) obtained a prevalence of 23.2% in the analysis of fecal samples from Eurasian badgers in Italy, using commercial immunoassay methods. As for the viverrids, to the authors’ knowledge, there are no other previous studies analyzing the presence of these enteroparasites in Europe. Outside of Europe there is a research detecting a 0.1% *Cryptosporidium* spp. infection rate in masked palm civets (*Paguma larvata*) from Southern China (71).

Some of the *Cryptosopiridum* spp. species detected in this study are related to the diet of the mesocarnivore species that tested positive (such as *C. erinacei* and *C. ditrichi* in Eurasian badger, *C*. sp. mouse genotype II in the red fox and *C. muris* in the common genet). This finding acts as supporting evidence for trophic chain transmission, aligning with previous studies (59). The diet of these carnivore species includes small mammals that can act as carriers of *Cryptosporidium* spp.; therefore, it is not surprising to observe species not specific to red foxes, badgers, or genets (65). Regarding *C. erinacei*, previous studies have shown that up to 30% of the European population of hedgehogs (*Erinaceus europaeus*) shed this parasite into the environment, therefore contributing to its spreading (72). Additionally, it has been previously detected in humans (73).

However, this raises the hypothesis of whether the presence of this parasite is due to the consumption of infected animals and the development of the infection (therefore making infected mesocarnivores to act as true hosts) or are simply mere vehicles of *Cryptosporidium* (i.e., no active infection). The available literature does not allow us to reach a solid conclusion regarding which of the two exposed hypotheses is more likely; however, recent research provides more evidence for the second scenario (59). What is undeniable is that the study of the dynamics of trophic interactions in the environment is fundamental.

Finally, regarding *Blastocystis*, we obtained a prevalence of 8.59%, making it the most prevalent parasite in this study. There is only one previous study in Spain that analyzes the presence of this enteroparasite in wild carnivores, which, showing a prevalence of 1.6%, is lower than the present work (28), suggesting that *Blastocystis* distribution across Spain is quite heterogenic and, therefore, of higher concern in our study area. Additionally, we observed significant differences in prevalence values between samples obtained from different provinces; mainly, prevalence values from Valencia (5.07%, 7/138) were higher than values from Castellón (19.23%, 5/26), Alicante (7.55%, 4/53). These results, however, have to be interpreted with caution, as Valencia is the province with the highest sample size (*N =* 138, compared to 26 from Castellón, 53 from Alicante, and only other 4 from unknown origin). Therefore, this disparity in prevalence values could be due to the higher biased sampling effort that happened in Valencia. There were also significant differences regarding the origin of samples, with samples coming from hunted animals being lower than the other groups (6.66%,1/15), while roadkill had the highest number of positive animals (8.33%, 12/144). But, again, the results have to be considered with caution, given the disparity in the sample size from each sample origin. Additionally, due to the very limited knowledge currently available, regarding the pathophysiology of *Blastocystis* sp., these results are very difficult to interpret.

*Blastocystis* subtypes that circulate among wild mesocarnivore populations in Spain are ST7 in common genet; ST14 in European mink; and ST1, ST2, ST4, ST7 and ST14 in red fox (28). In our study, we detected ST4 (*N =* 1) and ST15 (*N =* 1) in common genet; ST3 (*N =* 1), ST5 (*N =* 5), ST6 (*N =* 1), ST7 (*N =* 3) and ST15 (*N =* 2) in red foxes and ST15 (*N =* 1) in European mink. Additionally, we also found ST5 (*N =* 1) and ST15 (*N =* 1) in stone marten, ST5 (*N =* 1) in Eurasian badger and ST5 (*N =* 1) in the common weasel. Some of these subtypes have also been found in humans. To the authors’ knowledge, this study has analyzed the widest range of potential *Blastocystis* sp. hosts (nine mesocarnivore species) that can be found in Spain; and has also detected the enteroparasite in six out of nine species.

Recently, Figueiredo et al. (74) investigated the presence of *Blastocystis* in various wild ruminants across the Iberian Peninsula, reporting a prevalence of 13.8% using conventional PCR and next-generation amplicon sequencing. Results in carnivores are generally lower, as it is believed that carnivores are less prone to *Blastocystis* colonization (75). Moreover, the variety of *Blastocystis* subtypes tends to decrease as the host’s diet becomes more specialized (75), which would explain why a generalist carnivore such as the red fox shows the greatest diversity found in our study. A total of 15 *Blastocystis* subtypes (STs) were identified, including 14 previously known STs (ST2, ST5, ST10, ST13, ST14, ST21, ST23–ST26, ST30, and ST42–ST44), and one novel subtype, designated ST49.

Despite the apparent absence of strict host specificity among *Blastocystis* subtypes, comparative analysis with our data from wild mesocarnivores revealed overlap in only a single subtype, ST5, even though both host groups inhabit the same ecosystems. This observation suggests that environmental exposure alone does not fully account for the distribution of subtypes, and that host-related factors—such as differences in gut physiology, diet, immune response, or microbiome composition—may significantly influence host susceptibility and subtype establishment.

At a European level, presence of *Blastocystis* has been studied in red fox, wolf (*Canis lupus*), Eurasian badger and wild boar (*Sus scrofa*) in Slovakia, detecting its presence only in wild boar (76). Globally, in northeastern China they observed that the prevalence of this parasite was higher in birds (7%) than in wild mammals (3.8%) (77, 78).

Despite being a parasite that has caught the interest of the scientific community in recent years, there are still many unknown aspects of its pathogenesis and biological cycle. Furthermore, the number of studies to detect *Blastocystis* presence in wildlife and the potential transmission from wildlife to humans is still severely limited.

Interestingly, co-infections were also detected between different parasites and in different species; with the red fox being the source of two of the observed combinations (*G. duodenalis* and *Cryptosporidium* spp., and *Cryptosporidum* spp. and *Blastocystis* sp.). Although the studied parasites occupy different sections from the gastrointestinal tract, all of them are shed through their faeces and their main transmission route is faecal-oral or through the exposure of contaminated food and/or water sources. Therefore, these results can indicate exposure to environmental hotspots where multiple zoonotic agents circulate. Such co-infections may also influence parasite dynamics by increasing shedding intensity or prolonging infection, thereby enhancing the potential for environmental contamination. These findings reinforce the role of mesocarnivores as important reservoirs or bridge hosts within anthropized landscapes, where close spatial overlap with humans and domestic animals may facilitate multi-pathogen transmission under a One Health framework. Furthermore, the detection of the three parasite species in the red fox; coupled with being the species in our study that had the highest diversity of *Cryptosporidium* spp. species, and presented two of the co-infection combinations suggests that the red fox is the most important mesocarnivore species regarding zoonotic risk transmission potential (out of all the mesocarnivore species studied in this work). Thus, given its classification as a game species in Spain, could suggest that the red fox is a good candidate as a sentinel species to monitor these parasites prevalence in the environment.

## 5 Conclusion

It is confirmed that *Giardia duodenalis, Cryptosporidium* spp. and *Blastocystis* are present in wild mesocarnivore populations distributed throughout the Valencian Community (Eastern Spain), although their prevalence is low. *Giardia* is the most detected parasite, found in seven out of the nine species under investigation. Additionally, wild mesocarnivore species in Valencian Community act as hosts for *Cryptosporidium* spp. (*C. meleagridis, C. ditrichi, C. canis, C. erinacei, C. muris*) and diverse subtypes of *Blastocystis* sp. (ST3, ST4, ST5, ST6, ST7) with zoonotic potential, posing a risk to public health. Finally, wild mesocarnivores carry various species of *Cryptosporidium* spp. from the prey they consume. This finding emphasizes the importance of understanding not only the biology of the parasite but also the dynamics of trophic interactions in ecosystems.

## Data Availability

The datasets presented in this study can be found in online repositories. The names of the repository/repositories and accession number(s) can be found at: https://www.ncbi.nlm.nih.gov/nuccore/ accession numbers: PP002598 to PP002601, PP002606- PP002613.

## Footnotes

1
http://blast.ncbi.nlm.nih.gov/blast
